# Pitfalls in the use of sex chromosome sequence markers for internal quality control of next-generation sequencing

**DOI:** 10.17179/excli2024-7387

**Published:** 2024-09-05

**Authors:** Danielle Patchell, Stephen E. Langabeer

**Affiliations:** 1 Cancer Molecular Diagnostics, St. James's Hospital, Dublin, Ireland

## ⁯⁯

Next-generation sequencing (NGS) affords the simultaneous sequencing of multiple DNA regions of interest and is becoming widely adopted into the routine molecular diagnostic pathways of many pathology disciplines including hematology/oncology. Ongoing external quality assurance schemes and rigorous internal quality control (IQC) are necessary to ensure both reproducible and reliable results (Endrullat et al., 2016[4]). Among various levels of IQC processes is the inclusion and amplification of sex identification marker from both X and Y chromosomes. Sex typing of samples by co-amplification of a region of both AMELX and AMELY genes is a procedure widely used in forensic pathology (Nagare et al., 2018[8]).

At a central, molecular diagnostic laboratory for hematological and solid tumors, one application of NGS is the detection of driver mutations associated with the Philadelphia chromosome-negative myeloproliferative neoplasms. Using a single-molecule molecular inversion probe approach, variants within the four most commonly mutated exons (JAK2 exons 12 and 14, CALR exon 9 and MPL exon 10) are identified with co-amplification of a region of AMELX and AMELY genes also included (Eijkelenboom et al., 2016[3]; Lee Tokar et al., 2022[7]). From establishment of this assay, twelve cases have been identified where AMELX and AMELY sequence reads contradicted the gender provided on the request form (Supplementary Table 1). Further investigation revealed that nine patients had undergone hematopoietic allogeneic stem cell transplantation (HASCT) with a sex-mismatched sibling or unrelated donor and the three other cases were from male transgender patients who were undergoing or had completed male to female gender reassignment. 

These cases do not represent a failure of NGS IQC per se, but reinforce the requirement to investigate for the underlying genetic explanation (Hu et al., 2024[5]). In addition to HASCT from a sex-mismatched donor and gender reassignment as outlined above, other plausible confounders of this type of IQC include wrong blood in tube (Bolton-Maggs et al., 2015[1]) and patients with disorders of sex development including XY gonadal dysgenesis (Bumbuliene et al., 2022[2]). Given the increasing adoption of NGS technology into a number of pathology disciplines together with an increase in referrals to gender identity units, a shift in the assigned sex ratio of adolescents and a growing number of individuals with non-binary gender identities observed in this country and globally (Kearns et al., 2023[6]), greater awareness of this discrepancy is required.

## Declaration

### Acknowledgments

None.

### Conflict of interest

The authors declare no competing interests.

### Funding

The authors declare that no funds, grants or other support were received regarding preparation of this manuscript.

## Supplementary Material

Supplementary information
