# Supplementary material for: Pitfalls in the use of sex chromosome sequence markers for internal quality control of next-generation sequencing
Source: EXCLI J. 2024 Sep 5;23:1183–4. doi: 10.17179/excli2024-7387 (PMC11464863; doi:10.17179/excli2024-7387)
Supplement: Supplementary information [file EXCLI-23-1183-s-001.pdf]

EXCLI Journal 2024;23:1183-1184 – ISSN 1611-2156
Received: May 13, 2024, accepted: September 04, 2024, published: September 05, 2024

Supplementary information to:

Letter to the editor:

PITFALLS IN THE USE OF SEX CHROMOSOME SEQUENCE MARKERS FOR INTERNAL QUALITY CONTROL OF NEXT-GENERATION SEQUENCING

Danielle Patchell, Stephen E. Langabeer*

Cancer Molecular Diagnostics, St. James's Hospital, Dublin, Ireland

* **Corrresponding author:** Stephen E. Langabeer, Cancer Molecular Diagnostics, St. James's Hospital, Dublin D08 W9RT, Ireland, Tel: +353 1 4162413, E-mail: slangabeer@stjames.ie

https://dx.doi.org/10.17179/excli2024-7387

This is an Open Access article distributed under the terms of the Creative Commons Attribution License (http://creativecommons.org/licenses/by/4.0/).

EXCLI Journal 2024;23:1183-1184 – ISSN 1611-2156
Received: May 13, 2024, accepted: September 04, 2024, published: September 05, 2024

Supplementary Table 1: Cases with sex discrepancy detected by next-generation sequencing

Case	Age	Reason for MPN request	Gender on request form	NGS genotyping	Explanation
1	55	post-HASCT erythrocytosis	F	XY	ALL post-HASCT; male donor
2	56	*CALR*^{mut} MRD	F	XY	MDS/MPN post-HASCT; male donor
3	58	*CALR*^{mut} MRD	F	XY	PMF post-HASCT; male donor
4	60	*CALR*^{mut} MRD	F	XY	PMF post-HASCT; male donor
5	32	monocytosis, tear drop poikilocytes	M	XX	Ph+ ALL post-HASCT; female donor
6	54	post-HASCT erythrocytosis	M	XX	AML post-HASCT; female donor
7	54	*CALR*^{mut} MRD	M	XX	PMF post-HASCT; female donor
8	66	*JAK2*^{mut} MRD	M	XX	AML post-HASCT; female donor
9	57	*JAK2*^{mut} MRD	M	XX	PPV-MF post-HASCT; female donor
10	25	erythrocytosis	F	XY	Male>female transgender reassignment
11	47	thrombocytosis	F	XY	Male>female transgender reassignment
12	37	erythrocytosis	F	XY	Male>female transgender reassignment

MPN: myeloproliferative neoplasms; NGS: next-generation sequencing; HASCT: hematopoietic allogeneic stem cell transplant; ALL: acute lymphoblastic leukemia; MRD: measurable residual disease; MDS/MPN: myelodysplastic syndrome/myeloproliferative neoplasm; PMF: primary myelofibrosis; Ph+ ALL: Philadelphia chromosome-positive acute lymphoblastic leukemia; AML: acute myeloid leukemia; PPV-MF: post polycythemia vera-myelofibrosis
